# A versatile δ-aminolevulinic acid (ΑLA)-cyclodextrin bimodal conjugate-prodrug for PDT applications with the help of intracellular chemistry

**DOI:** 10.3762/bjoc.10.251

**Published:** 2014-10-17

**Authors:** Chrysie Aggelidou, Theodossis A Theodossiou, Antonio Ricardo Gonçalves, Mariza Lampropoulou, Konstantina Yannakopoulou

**Affiliations:** 1 Institute of Advanced Materials, Physicochemical Processes, Nanotechnology & Microsystems, National Center for Scientific Research “Demokritos”, Patriarchou Gregoriou & Neapoleos, Aghia Paraskevi Attikis, 15310 Greece. Tel. +30210 6503796, Fax: +30 210 6511766

**Keywords:** cyclodextrins, PDT, protoporphyrin IX, prodrug, δ-aminolevulinic acid

## Abstract

Grafting of δ-aminolevulinic acid (**1**) moieties on the narrow periphery of a β-cyclodextrin (β-CD) derivative through hydrolysable bonds was implemented, in order to generate a water-soluble, molecular/drug carrier with the capacity to undergo intracellular transformation into protoporphyrin IX (PpIX), an endogenous powerful photosensitizer for photodynamic therapy (PDT). The water-soluble derivative **2** was prepared by esterifying δ-azidolevulinic acid with heptakis(6-hydroxyethylamino-6-deoxy)-β-cyclodextrin, with an average degree of substitution, DS = 3. Delivery of water-soluble, colorless **2** to cells resulted in intense red fluorescence registered by confocal microscopy, evidently due to the engagement of the intracellular machinery towards formation of PpIX. Conjugate **2** was further complexed with a fluorescein-labeled model guest molecule which was successfully transported into the cells, thereby demonstrating the bimodal action of the derivative. The present work shows the versatility of CDs in smart applications and constitutes advancement to our previously shown PpIX-β-CD conjugation both in terms of water solubility and lack of aggregation.

## Introduction

**Scheme 1:**
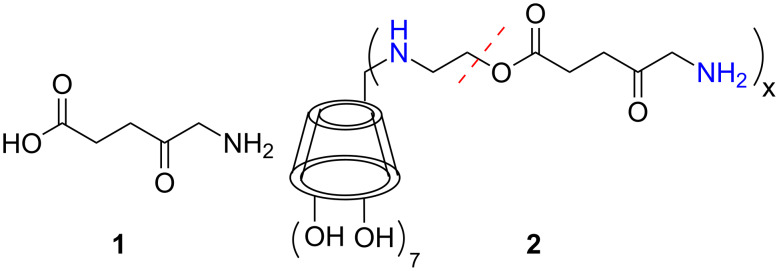
The structure of δ-aminolevulinic acid (**1**), a precursor in cellular biosynthesis of protoporphyrin IX and subsequently heme.

Porphyrins have long been used as agents for tumor photodiagnosis (PDD) and photodynamic therapy (PDT) because of their preferential accumulation in cancerous rather than squamous tissues [1]. δ-Aminolevulinic acid (**1**, ALA, Scheme 1) is a δ-amino acid precursor in the cellular biosynthesis of heme [2].

**Scheme 2:**
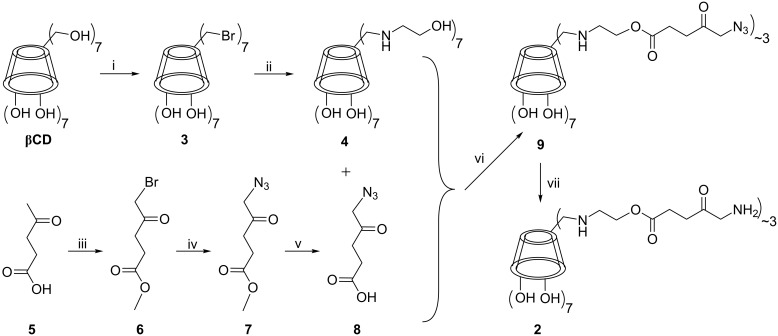
Synthesis of conjugate **2** i) Br_2_, Ph_3_P, DMF, 75–80 °C, 93% according to [16]; ii) 2-hydroxyethylamine, 80 °C, 76%, according to [12]; iii) Βr_2_, MeOH, rt, 41%; iv) NaN_3_, THF, 25–30 °C, 90%; v) phosphate buffer, pig liver esterase, pH 8, 25–30 °C, 82%; vi) DCC, DMAP, DMF, 25–30 °C, 53%; vii) H_2_, Pd/C, 25–30 °C, 95%.

The penultimate step in this biosynthetic cycle is the iron chelation of protoporphyrin IX (PpIX), a porphyrin photosensitizer (PS) which was shown to be very efficient for PDT [3]. Compound **1** and its more lipophilic esters, methyl and hexyl levulinate, have been approved for clinical use as PpIX precursors against several cancers such as basal cell carcinoma, bladder cancer, actinic keratosis, etc. [4]. Compound **1** is a polar, zwitterionic compound, with inherently poor cell permeability and consequently challenging pharmaceutical formulations [5]. We have recently published [6] the covalent conjugation of PpIX to β-cyclodextrin (β-CD), a water-soluble cyclic oligosaccharide host capable of carrying hydrophobic molecules, such as drugs, in its hydrophobic cavity [7]. The cyclodextrins (CDs) are a notable family of semi-natural carbohydrate molecules approved as pharmaceutical excipients that improve the solubility and bioavailability of drugs through molecular encapsulation. We demonstrated [6] the conjugate’s (PpIX-CD) bimodal action of transporting the chemotherapeutic agent tamoxifen intracellularly to engage its targets while simultaneously acting as a PS. In this sense we created a bimodal system with adequate water solubility (several μM) able to inflict significant chemo- and phototoxicity on cancer cells. One additional benefit of tethering β-CD to PpIX, was the reduced aggregation of the conjugate in aqueous solutions. Formation of aggregates is an adverse yet typical characteristic of porphyrins that reduces their photodynamic efficiency, quenches their fluorescence and complicates their delivery [8–10]. In the system presented herein, the problems of limited aqueous solubility as well as that of aggregation are completely bypassed, as multiple copies of **1** are attached to a β-CD derivative bearing hydroxyethylamino groups in all seven positions of the narrow opening. Τhe resulting conjugate (Scheme 1, **2**) will encompass (i) the wide β-CD opening available for inclusion of a hydrophobic guest molecule for intracellular transport, (ii) positively charged secondary and primary amino groups at physiological pH thus able to interact with negatively charged components of the cell membranes and achieve cell penetration [11] and (iii) cleavable ester bonds expected to release **1** by the action of intracellular enzymes. Taken together, the strategy would combine prodrug photosensitizer capability with molecular/drug transport in a single versatile bimodal system.

## Results and Discussion

**Synthesis*:*** The suitable β-CD derivative to be linked with ALA (**1**) was heptakis(6-hydroxyethylamino-6-deoxy)-β-CD (**4**, Scheme 2), obtained from heptakis(6-bromo-6-deoxy)-β-CD **3** and ethanolamine in high yield [12]. The choice of **4** was based on the presence of readily accessible primary terminal hydroxy groups on its narrow side, facilitating the formation of hydrolysable ester bonds with **1**. The resulting conjugate **2** (Scheme 1), expected to be water soluble, comprises seven secondary and multiple primary (maximum seven) amino groups in the final form. Initial attempts to directly couple compound **1** with the terminal hydroxy groups in **4** consistently failed due to the very high propensity of **1** to reversibly lactonize or dimerize into dihydropyrazine derivatives (DHPY), which then irreversibly aromatize to pyrazines [13] thus resulting in various side products that impede purification. Protection of the δ-amino group in **1** with the fluorenylmethyloxycarbonyl (Fmoc) group did not eventually lead to coupling products with **4**. Furthermore, benzyloxycarbonyl (Ζ) protection of **1** and subsequent successful coupling reaction with **4** gave the desired Z**-**protected coupling product which, however, resisted removal of the Z protecting group under a variety of conditions [(i) ΜeOH, Pd/C, H_2_, (ii) ΜeOH, formic acid, Pd/C, H_2_, (iii) ΜeOH, acetic acid, Pd/C, H_2_, (iv) ΜeOH, trifluoroacetic acid, Pd/C, H_2_]. A successful alternative was subsequently adopted comprising the linking of **4** to δ-azidolevulinic acid (**8**) (Scheme 2). The latter, having the nucleophilicity of the δ-position silenced, presented a completely straightforward precursor to **1** and proved to be amenable to coupling with **4** under various reaction conditions. Briefly [14], **8** was prepared from levulinic acid (**5**) through δ-bromination and concomitant methyl ester formation (compound **6**), followed by azidation to **7**. Finally, enzymatic cleavage of the ester [15] resulted in liberation of the carboxylic acid functionality that is required for the coupling to the CD (Scheme 2, compound **8**, Figure S1, Figure S2 in Supporting Information File 1).

The connection of **8** to **4** proceeded under standard coupling conditions (DCC, DMAP) [17] affording a water-soluble compound. The ^1^H NMR spectrum of the product verified the presence of peaks arising both from **8** chain and **4** components, while the relative broadness of the signals indicated incomplete/random substitution, allowing, however, the assignment of peaks via the HSQC-edited spectrum (Supporting Information File 1, Figure S3). The ratio **8** to **4** was ~3, as indicated by integration of the respective signals and also confirmed by MALDI–TOF MS measurements (*m*/*z* 1853, Supporting Information File 1, Figure S4), whereas the IR spectrum exhibited the characteristic stretching vibrations of the azido group (2104 cm^−1^, (Supporting Information File 1, Figure S5). The so obtained conjugate **9** was subsequently reduced under strongly acidic catalytic hydrogenation conditions to **2** in nearly quantitative yield.

**Figure 1:**
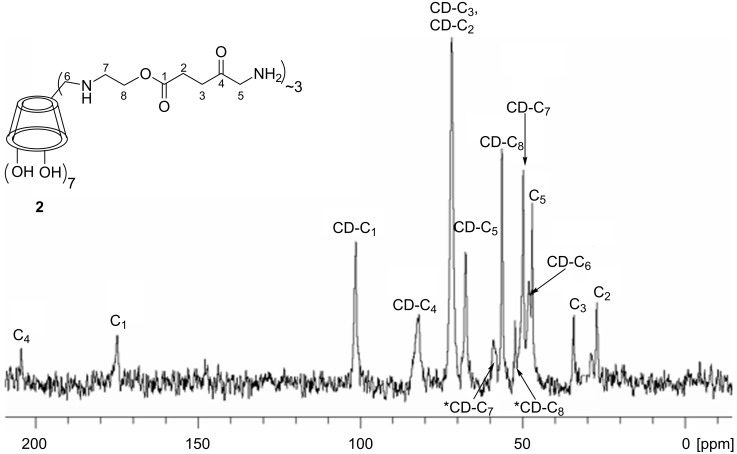
^13^C NMR spectrum of **2** (D_2_O, 125 MHz) with assignment of the peaks.

Crucial to the spectroscopic characterization of the product was the ^13^C NMR spectrum (Figure 1) where the ester and ketone carbonyl signals were observed along with the signals of the β-CD moiety. Moreover, in the IR spectrum of **2** (Supporting Information File 1, Figure S5) the azido-group band disappeared, whereas prominent peaks were observed in the carbonyl region. Finally the mass spectrum confirmed the identity of the product, which maintained an average molecular weight corresponding to ~3 δ-aminolevulinic acid moieties attached.

**Figure 2:**
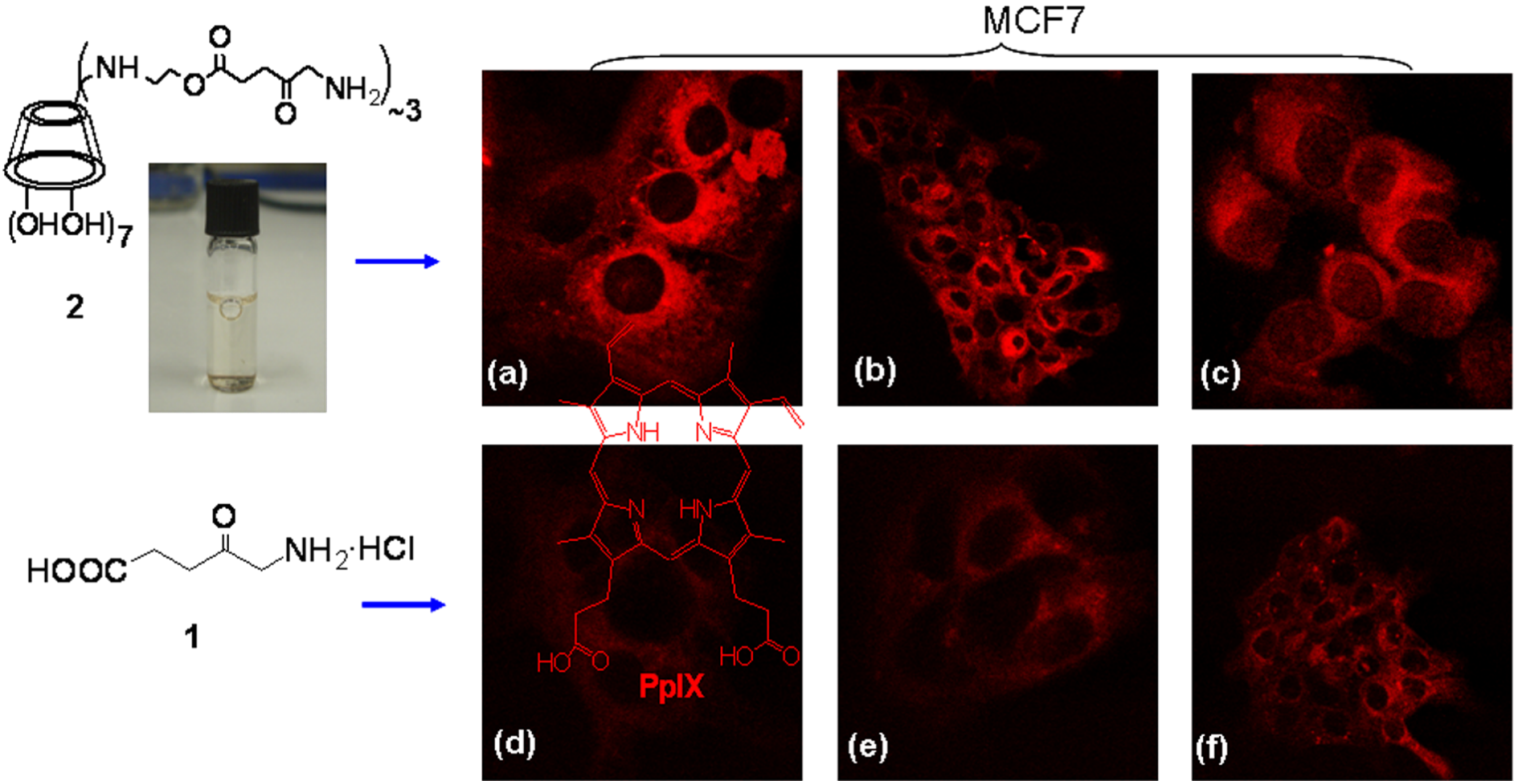
Representative confocal microscopy images of MCF7 cells incubated with 1 mM **2** (a, b and c), and 1 mM **1** (d, e, f), (λ_ex_ = 568 nm, λ_em_ > 585 nm). The laser intensity used for the acquisition of images a, b, c was 30% of that used for images d, e and f.

**Cell studies with 2:** The capability of the conjugate to cross cell membranes and to produce PpIX intracellularly was examined. MCF7 cells were incubated with the colorless conjugate solution for 4 h, to allow conversion of prodrug **1** into PpIX [5,18]. Indeed, upon confocal microscopy imaging of the treated cells (λ_ex_ = 568 nm), intense red fluorescence was observed (Figure 2), demonstrating that either (i) cell internalization was accompanied by esterolysis and release of **1** which was subsequently converted into PpIX, or (ii) one molecule of **2** with five molecules of **1** produced PpIX-(mono)CD or (iii) two molecules of **2** interacted with two molecules **1** to form PpIX-(bis)CD. The first of these three scenarios is the most probable given the abundance of intracellular esterases, which would engage into activity as with the approved and marketed lipophilic esters of **1** [5], however, the other two possibilities cannot be excluded. The red fluorescence of cells incubated with **2**, evidently originating from PpIX formation, was more intense in comparison with the flurorescence from cells treated with the same concentration of **1**·HCl. Specifically, images taken from cells that were treated with either **1** or **2** under identical conditions were analyzed with regard to the intensity of red color with the MATLAB software. 2D analysis and superimposition of the 1D images along the *x*-axis (Supporting Information File, Figure S7) revealed that the intensity of red fluorescence was measurably higher in both images taken from cells after treatment with **2** than in those treated with **1**, irrespective of the laser intensity applied. Incubation with compound **1** and use of 100% laser intensity resulted in only small enhancement of red fluorescence.

These qualitative results show that the conjugate **2** worked much more efficiently than **1** alone, either due to the multiplicity of **1** attached and/or due to more efficient cell penetration. What can be, however, deduced from the images in Figure 2 and Figure S7 is that although the **1**-treated cells were imaged at more than 3-times the laser power used for the **2**-treated cells, the latter exhibited a much more intense PpIX fluorescence. This indicates that it is not the 3×δ-aminolevulinic acid molecular multiplicity of **2** that is solely responsible for the PpIX fluorescence enhancement, but either an augmented cell entry or the inhibition of ferrochelatase-catalyzed iron insertion to form non-fluorescent iron-complexed heme [19] (possibly due to the existence of PpIX-CD moieties). These suggestions as well as quantitation of subcellular processes (hydrolysis rate of **1**, PpIX concentration) could only be confirmed with specific mechanistic biological studies, which lie outside the scope of the present work.

**Figure 3:**
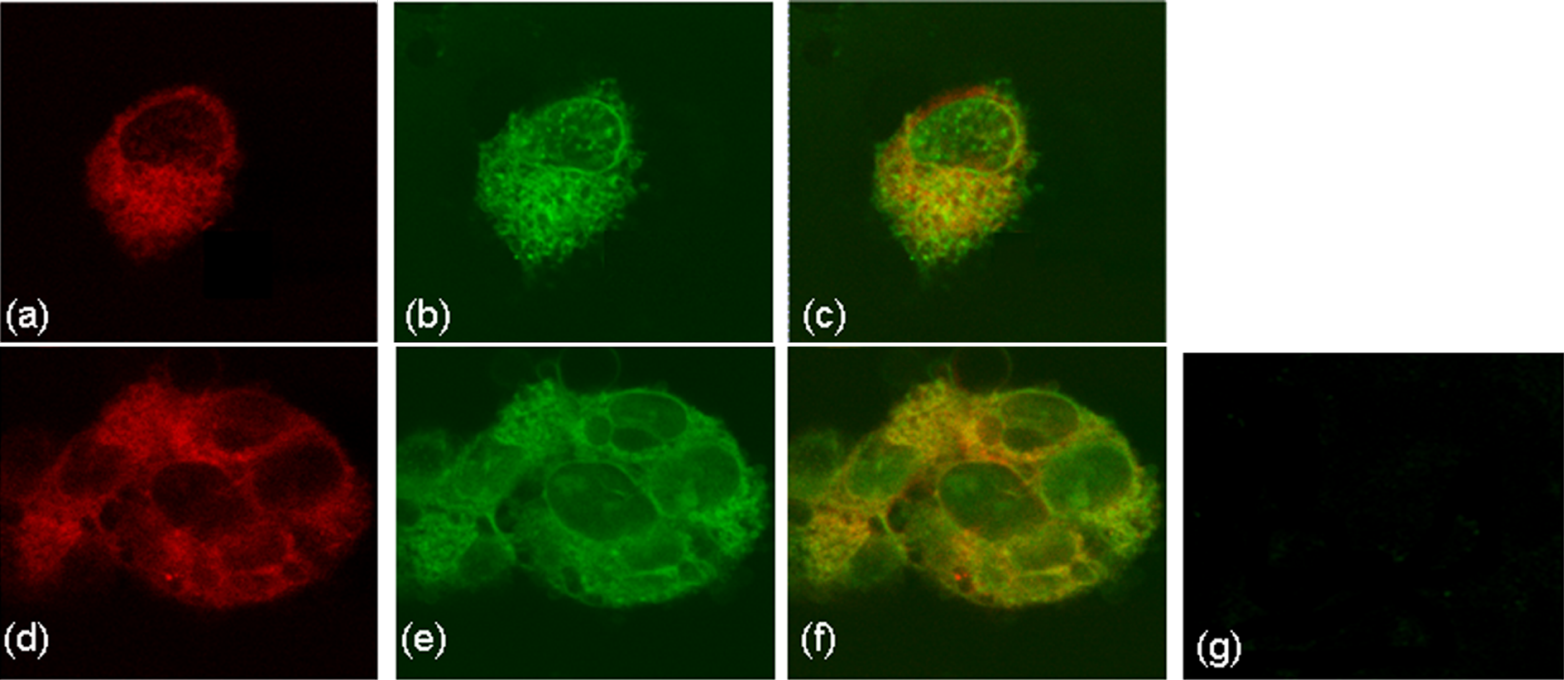
Representative confocal fluorescence microscopy images of MCF7 cells after incubation with a solution of **2**–fluorescein isothiocyanate-labeled 1-adamantylamine (1 mM/0.2 mM) PBS: (a, d) red fluorescence (λ_ex_ = 568 nm, λ_em_ > 585 nm); (b, e) green fluorescence (λ_ex_ = 488 nm, λ_em_ = 522 nm); (c, f) overlay of images a, b and of d, e: yellow color indicates guest co-localization with PpIX core; (g) blank experiment of fluorescein isothiocyanate-labeled 1-adamantylamine (0.2 mM in PBS, λ_ex_ = 488 nm, λ_em_ = 522 nm ).

We further incubated MCF7 cells with **2** pre-complexed with water-insoluble fluorescein isothiocyanate-labeled 1-adamantylamine (Figure 3). The characteristic confocal microscopy images revealed red fluorescence upon excitation at λ_ex_ = 568 nm, as well as green fluorescence upon excitation at 488 nm. Following superimposition of the two fluorescence channels it could be concluded that PpIX production and CD guest intracellular localization, largely coincided within the cell cytoplasm (Figure 3). This indicates that possibly some of the levulinic acid ester moieties in **2** not have undergone enzymatic cleavage or hydrolysis but instead have proceeded as a conjugate through the heme biosynthesis, resulting in the biosynthesis of PpIX-CD species. This hypothesis, however, can only be confirmed by using **1**-CD species, bearing no cleavable tethers to the cyclodextrin core. Moreover, is well known that β-CDs affect cells by interacting with their membrane components, namely depleting and enriching cholesterol in lipid raft and non-raft regions as well as removing and redistributing phospholipids [20]. Thus, the transport of an adamantyl-substituted guest that blocks the β-CD cavity is anticipated to alter the above host–membrane interactions. In this respect, the primary side enrichment of **1**-CD with amino groups is expected to play a major role in the interaction with the anionic parts of phospholipids, rather than the β-CD cavity and its encapsulation ability of hydrophobic membrane components.

## Conclusion

The present work demonstrates the synthesis and characterization of the bimodal conjugate prodrug **2**, bearing ~3 δ-aminolevulinic acid moieties per β-CD macrocycle. The dual nature of the system was exemplified by the intracellular formation of PpIX using “intracellular chemical synthesis”. At the same time **2** was able to carry inside the cytoplasm a model guest molecule. The possibility that the intracellular biosynthetic machinery may have produced some PpIX-CD species cannot be ruled out. Overall, successful δ-aminolevulinic acid intracellular delivery with complete aqueous solubility, PpIX production and transport of a model guest were achieved.

## Experimental

**Ester of δ-azidolevulinic acid with heptakis(6-hydroxyethylamino-6-deoxy)-β-cyclodextrin (9).** Heptakis(6-hydroxyethylamino-6-deoxy)-β-cyclodextrin (**4**, 62 mg, 0.043 mmol) was dissolved in dimethylformamide (DMF, 2 mL), cooled to 0 °C and stirred for 15 min. Then, 4-dimethylaminopyridine (DMAP, 3.7 mg, 0.0302 mmol) and *Ν,Ν*΄-dicyclohexylcarbodiimide (DCC, 68.6 mg, 0.332 mmol) were added, followed by δ-azidolevulinic acid (**8**, 52.2 mg, 0.332 mmol), which had previously been stirred in DMF (1 mL) for 6 h in the presence of a KOH pellet. The mixture was stirred at rt for 48 h under argon. Then it was centrifuged to remove insoluble side product dicyclohexylurea and the supernatant was collected and condensed to dryness. The solid residue was dissolved in a small volume of water and dialyzed from a benzoylated cellulose membrane (MW_CO_ ~2000) for 24 h. The solvent of the dialyzed solution was evaporated and the residue was lyophilized, to afford an off-white solid with degree of substitution ~3, (53%). ^1^H ΝΜR (500 ΜHz, D_2_O, 298 K) δ 5.16 (brs, 7H, CD-H1), 4.28 (s, 5H, H5), 3.96 (brs, 14H, CD-H3, CD-H5), 3.78 (brs, 14H, CD-H8), 3.67 (brs, 14H, CD-H2, CD-H4), 3.36–2.77 (m, 28H, CD-H6, CD-H6΄, CD-H7), 2.72 (t, *J* = 7.0 Hz, 5H, H2), 2.47 (t, *J* = 7.0 Hz, 5H, H3), 2.13–1.06 (m, 1,3-dicycloxylurea byproduct) ppm; ^13^C NMR (62 ΜHz, CDCl_3_, 298 K) δ 207.4 (C4), 183.1–173.4 (C1), 101.7 (br, CD-C1), 82.91 (br, CD-C4), 71.3, 72.0, 69.9 (CD-C3, CD-C5, CD-C2), 58.7 (CD-C8), 57.2 (C5), 50.29, (CD-C7), 48.70 (CD-C6,6΄), 31.1 (C3), 27.0 (C2), 36.0–22.7 (1,3-dicycloxylurea byproduct) ppm; IR ν: 2104.2 (vs) cm^−1^; MS (MALDI–TOF) *m*/*z*: 1853.7 (100%, [M + Η]^+^); Exact mass calculated for C_71_H_120_N_16_O_41_ (**9**): 1852.78.

**Ester of δ-aminolevulinic acid with heptakis(6-hydroxyethylamino-6-deoxy)-β-cyclodextrin (2).** To a solution of **9** (20 mg, 0.0112 mmol) in ethanol (1 mL), CF_3_COOH (250 μL) was added dropwise followed by addition of Pd/C (7.5 mg, 0.0071 mmol, 0.06 equiv). The mixture was hydrogenated with Η_2_ gas under stirring at rt for 48 h. Subsequently the catalyst was removed by repeated centrifugation cycles and the solution was evaporated to dryness, the residue re-dissolved in water and dialyzed as above. The resulting solution was evaporated and the residue lyophilized to obtain the desired solid product (95%). ^1^H ΝΜR (500 MHz, D_2_O, 298 K) δ 5.18 (brs, 7H, CD-H1), 4.13 (s, 5H, H5), 3.98 (brs, 14H, CD-H3, CD-H5), 3.89 (brs, 14H, CD-H8), 3.78–3.57 (m, 14H, CD-H2, CD-H4), 3.30 (brs, 28H, CD-H6, CD-H6΄, CD-H7), 2.92 (t, *J* = 6.5 Hz, 5H, H2), 2.73 (t, *J* = 6.5 Hz, 5H, H3) ppm; ^13^C NMR (62 MHz, CDCl_3_, 298 K) δ 204.5 (C4), 175.2 (C1), 101.7 (CD-C1), 82.3 (br, CD-C4), 71.8, 67.6 (CD-C3, CD-C5, CD-C2), 59.1, 56.5 (CD-C8, CD-C8), 52.5, 49.9 (CD-C7, CD-C7), 48.11 (CD-C6,6΄), 34.4 (C3), 27.1 (C2) ppm; MS (MALDI–TOF) *m*/*z*: 1685.2 (100%, [M + Νa]^+^), 1798.18 (89%, [M’ + Na]^+^); exact mass calculated for C_66_H_119_N_9_O_39_ ((**1**)_2_-CD **=** M): 1661.76 and for C_71_H_126_N_10_O_41_ ((**1**)_3_-CD = M’): 1774.808.

**Complexation of fluorescein isothiocyanate-labeled 1-adamantylamine with 2**. The conjugate, **2** (1 mM solution, with respect to ALA content) in phosphate buffer saline (pH 7.4) was mixed with fluorescein isothiocyanate-labeled 1-adamantylamine (0.2 mM) [21] and the dispersion was stirred for 20–30 min until a clear solution was obtained. This solution was subsequently used for cell incubation.

**Cell culture:** For the purposes of this study we chose the MCF7 human breast adenocarcinoma cell line. The cells were grown in RPMI 1640 without phenol red, supplemented with 10% FBS, penicillin/streptomycin at 37 °C in a 5% CO_2_ and 95% humidified atmosphere

**Confocal Microscopy:** MCF7 cells were seeded on 22 mm glass coverslips in 35 mm dishes (1 × 10^5^ cells per dish) 24 h prior to the experiments. The cells were subsequently incubated with 1 mM of **1** or **2** or alternatively **2** (1 mM)/fluorescein isothiocyanate-labeled 1-adamantylamine (0.2 mM) for 4 h. Afterwards the cells were washed with PBS and mounted on microscope slides for confocal fluorescence live-cell imaging performed as described previously [21]. Intracellular fluorescein isothiocyanate-labeled 1-adamantylamine was excited using the 488 nm line of an argon–krypton ion laser while the PpIX fluorescence was excited with the 568 nm line of the same laser. Fluorescein isothiocyanate-labeled 1-adamantylamine fluorescence was collected through a band-pass filter centered at 522 (± 35) nm, while PpIX fluorescence was collected after a long-pass filter at ≥585 nm. In a control experiment cells were incubated for 4 h with 0.2 mM ADA-FITC in PBS.

## Supporting Information

File 1 Full experimental procedures and detailed analytical data for the synthesis of all precursor molecules of Scheme 1; additional NMR, IR and mass spectral data.
