# Supplementary material for: A versatile δ-aminolevulinic acid (ΑLA)-cyclodextrin bimodal conjugate-prodrug for PDT applications with the help of intracellular chemistry
Source: Beilstein J Org Chem. 2014 Oct 17;10:2414–20. doi: 10.3762/bjoc.10.251 (PMC4222291; doi:10.3762/bjoc.10.251)
Supplement: File 1 — Full experimental procedures and detailed analytical data for the synthesis of all precursor molecules of Scheme 1; additional NMR, IR and mass spectral data. [file Beilstein_J_Org_Chem-10-2414-s001.pdf]

# Supporting Information

# for

# A versatile δ-aminolevulinic acid (ALA)-cyclodextrin bimodal conjugate-prodrug for PDT applications with the help of intracellular chemistry

Chrysie Aggelidou, Theodossis A. Theodossiou*[§], Antonio Ricardo Gonçalves, Mariza Lampropoulou, Konstantina Yannakopoulou*

Address: Institute of Advanced Materials, Physicochemical Processes, Nanotechnology & Microsystems, National Center for Scientific Research “Demokritos”, Patriarchou Gregoriou & Neapoleos, Aghia Paraskevi Attikis, 15310 Greece. Tel. +30210 6503796, Fax: +30 210 6511766

Email: Konstantina Yannakopoulou - dyanna@chem.demokritos.gr

*Corresponding authors

[§]Current address: Department of Radiation Biology, Institute for Cancer Research, The Norwegian Radium Hospital, Montebello, 0379 Oslo, Norway

**Full experimental procedures and detailed analytical data for the synthesis of all precursor molecules of Scheme 1; additional NMR, IR and mass spectral data**

# Experimental details

## Heptakis(6-bromo-6-deoxy)-β-cyclodextrin (**3**)

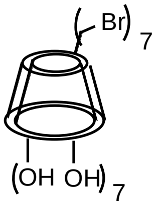


In a water bath at room temperature, dry dimethylformamide (DMF, 100 mL) was added to a flask equipped with a drying tube (KOH) and was vigorously stirred. Triphenylphosphine ($PPh_3$, 39.4 g, 0.15 mol) was added to this solution in portions and a temperature drop (25 ºC to 17 ºC) was noticed. *N*-bromosuccinimide (NBS, 28.6 g, 0.16 mol, light brown solid) was added in portions, while the temperature was kept bellow 40 ºC by addition of ice to the water bath. The colour of the solution changed to dark red and its viscosity increased, which was controlled by addition of DMF and vigorous stirring. β-cyclodextrin (βCD, 11.4 g, 0.01 mol) was added to the mixture (temperature increased) and the colour changed to dark green. Afterwards the solution was transferred to an oil bath (70 ºC – 80 ºC) and the reaction progress was monitored by TLC (silica gel 60, dioxane:ammonia:*i*PrOH (10:7:3), v/v). After the completion of the reaction, the mixture was cooled in a water bath for 10 min until the temperature dropped to 40 ºC – 50 ºC. Methanol (50 mL) was added and the solution was poured into a large excess of methanol (500 mL). The pH of the methanolic solution was recorded ($2 < pH < 3$, universal paper) and adjusted to $7 < pH < 8$ (universal paper) with addition of sodium methoxide ($CH_3ONa$, 3.21 g). The mixture was stirred for 5 min and left to stand for 10 to 15 min. A light brown solid precipitated from the methanolic solution that was extensively washed with more methanol and water to remove of over-brominated and under-brominated products, respectively, checked by TLC. The product, heptakis(6-bromo-6-deoxy)-β-cyclodextrin (**3**) was obtained as a light yellow powder (94.3 %, 14.9 g). $^1$H NMR (DMSO-$d_6$, 500 MHz, 24 ºC): δ (ppm) = 6.04 (d, $J$ = 7.0 Hz, 2-OH, 7H), 5.91 (br s, 3-OH, 7H), 4.98 (d, $J$ = 3.0 Hz, $H_1$, 7H), 4.01 (d, $J$ = 10.0 Hz, $H_6$, 7H), 3.83 (t, $J$ = 8.5 Hz, $H_5$, 7H), 3.66 (q, $H_3$, $H_{6'}$, 14H), 3.48 – 3.35 (m, $H_2$, $H_4$, 14H). $^{13}$C NMR (DMSO-$d_6$, 125 MHz, 24 ºC): δ (ppm) = 102.1 ($C_1$), 84.6 ($C_4$), 72.3 ($C_3$), 72.1 ($C_2$), 71.0 ($C_5$), 34.5 ($C_6$), in agreement with literature [1].

## Heptakis(6-hydroxyethylamino-6-deoxy)-β-cyclodextrin (**4**)

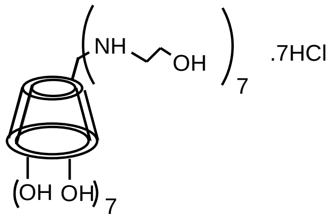


Heptakis(6-bromo-6-deoxy)-β-cyclodextrin (**3**, 0.200 g, 127 mmol) was dissolved in a large excess of distilled ethanolamine (1.5 mL) and a clear light brown solution was obtained, that was heated at 80 ºC for two days under argon (Ar) with periodic temperature checks (80 – 85 ºC). The excess ethanolamine was evaporated under vacuum (60 ºC), the oily crude residue was dissolved in water (1 mL) and acetone (100 mL) was added to precipitate the product. The solid obtained was redissolved in water (3 mL) and the pH was adjusted to 7 by addition of hydrochloric acid (HCl, 1 M). The solution was dialyzed using benzoylated cellulose dialysis tubing ($MW_{CO}$ ~ 2000) for three days. The product obtained after evaporation of the solvent was re-dissolved in water and sodium hydroxide was added to pH =10. The solution was treated with ion exchange resin (Amberilite, IR 120, strongly acidic, $H^+$) to remove traces of ethanolamine. Removal of the solvent and drying of the product at 60° C for 18 h afforded a white solid (**4**, 0.142 g, 66 %). $^1$H NMR ($D_2O$, 500 MHz, 24 ºC): δ (ppm) 5.26 (br s, 7H, $H_1$), 4.36 (t, *J* = 8.0 Hz, 7H, $H_5$), 4.06 (t, *J* = 8.8 Hz, 7H, $H_3$), 3.96 (br s, 14H, $H_8$), 3.75-3.67 (m, 14H, $H_2/H_4$), 3.57 (br d, 7H, $H_{6'}$), 3.50 - 3.46 (m, 7H, $H_6$), 3.38 – 3.34 (m, 7H, $H_8$). $^{13}$C NMR ($D_2O$, 125 MHz, 24 ºC): δ (ppm) 101.2 ($C_1$), 82.1 ($C_4$), 72.2 ($C_3$), 71.8 ($C_2$), 65.7 ($C_5$), 56.8 ($C_8$), 49.9 $C_7$), 48.4 ($C_6$) in agreement with the literature [1,2].

## Methyl 5-bromolevulinate (**6**) [3]

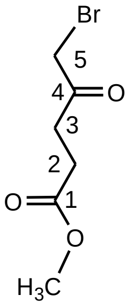


Levulinic acid (**5**, 11.34 g, 97.65 mmol) was added to methanol (MeOH, 98 mL) and stirred for 15 min in an ice-bath (0 ºC). Bromine (8.8 mL, 342 mmol, 2 eq.) was added dropwise for 3 h to the LA methanolic solution and the temperature was kept below 30 $^0$C. The reaction was left to stir for 12 h at room temperature (25 ºC) protected from light. The reaction mixture was then heated to reflux for 90 min. The reaction was then stopped by evaporating methanol under vacuum (30 ºC) and the residue obtained was dissolved in

dichloromethane ($CH_2Cl_2$, 45 mL), washed with water (45 mL) and with aq. sat. hydrogen carbonate ($NaHCO_3$, 25 mL) and again with water (3 x 25 mL). The organic phase was dried with magnesium sulphate ($MgSO_4$), the solvent was evaporated and the residue (yellow liquid) was stored in the freezer. Methyl 5-bromolevulinate (LA-Br) was isolated by flash column chromatography using silica gel (230-400 mesh) and a mixture of n-hexane/$CH_2Cl_2$ (v/v) (1:3.5) as mobile phase, and **6** was obtained as a yellow liquid (8.36 g, 41 %). $^1$H NMR ($CDCl_3$, 500 MHz, 24 ºC): δ (ppm) 3.95 (s, $H_5$, 2H), 3.67 (s, -$CH_3$, 3H), 2.94 (t, J = 6.5 Hz, $H_3$, 2H), 2.64 (t, J = 6.5 Hz, $H_2$, 2H). $^{13}$C NMR ($CDCl_3$, 125 MHz): δ (ppm) 200.7 ($C_4$), 172.9 ($C_1$), 52.1 (-$CH_3$), 34.5 ($C_3$), 34.2 ($C_2$), 28.2 ($C_5$). IR (cm$^{-1}$) = 3000, 2953, 2849 (var, aliphatic CH stretch); 1726 (str, C=O stretching); 1202 (str, $CH_2$-Br wagging); 1172 (C-CO-C stretch and bend) in agreement with the literature data [3]..

## Methyl 5-azidolevulinate (**7**) [3]

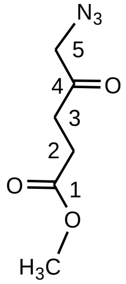


Sodium azide ($NaN_3$, 0.27 g, 4.2 mmol) was solubilised in deionized water (1 mL) in an ice-bath (0 °C) and a solution of methyl-5-bromolevulinate (**6**, 0.436 g, 2.08 mmol) in dry tetrahydrofuran (THF, 1.1 mL) was added dropwise. The mixture was stirred for 10 min at 0 ºC and then, brought to room temperature (25 ºC) and stirred for 1 h more. The reaction was stopped and the residue was purified by liquid-to-liquid extraction, performed using ethyl acetate (EtOAc, 200 mL) and water ($H_2O$, 200 mL). The organic layer was isolated, dried with anhydrous magnesium sulphate ($MgSO_4$, 10 g) and evaporated to give **7** as a yellow liquid (0.326 g, 91% yield) stored in the freezer. Experimental data agreed with literature [3]. $^1$H NMR ($CDCl_3$, 500 MHz, 24 ºC): δ (ppm) 4.00 (s, $H_5$, 2H), 3.65 (s, -$CH_3$, 3H), 2.80 – 2.50 (m, $H_3$/$H_2$, 4H). $^{13}$C NMR ($CDCl_3$, 125 MHz): δ (ppm) 203.1 ($C_4$), 172.8 ($C_1$), 57.4 (-$CH_3$), 52.0 ($C_5$), 34.3 ($C_3$), 27.5 ($C_2$). IR (cm$^{-1}$) = 3022, 2954 (aliphatic CH str); 2103 (str, azido); 1726 (str, C=O stretching); 1171 (var, C-CO-C stretch and bend) in agreement with the literature [3].

## 5-Azidolevulinic acid (**8**) [3]

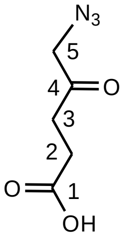

Methyl 5-azidolevulinate (**7**, 0.195 g, 1.14 mmol) was dispersed in phosphate buffer (0.1 M, pH=8, 4 mL). Pig liver esterase (PLE, 5.6 mg, 17 U/mg) was added to mixture and the solution was stirred at room temperature (25 ºC) for 1h. The progress of the reaction was followed by thin layer chromatography (TLC, silica gel 60 F254) using a mixture of dichloromethane/n-hexane (1:1, v/v). **7** appeared as a yellow spot (Rf = 0.54) whereas **8** did not progress in the TLC (Rf = 0), appearing as an orange spot). The pH of the solution was maintained at 8 with careful addition of NaOH (5 M). After 24 h the starting material had been consumed and the reaction was extracted with ethyl acetate (3 x 15 mL); the aqueous phase was acidified to pH = 2 with hydrochloric acid (HCl, 6 M) and was further extracted with EtOAc (3 x 20 mL). All organic phases were collected, washed with sodium chloride saturated solution (20 mL), followed by water ($H_2O$, 20 mL), and dried over anhydrous magnesium sulphate ($MgSO_4$). After evaporation the product was triturated with diethyl ether at 0 ºC and air-dried, to obtain **8** as a pale brown solid (0.147 g, 82 %). $^1$H NMR ($CDCl_3$, 500MHz, 24 ºC): δ (ppm) 9.55 (vb, -$CO_2H$, 1H), 4.00 (s, $H_5$, 2H), 2.73 (s, $H_2$/$H_3$, 4H). $^{13}$C NMR ($CDCl_3$, 125 MHz, 24 ºC): δ (ppm) 202.9 ($C_4$), 178.1 ($C_1$), 57.4 ($C_5$), 34.1 ($C_3$), 27.5 ($C_2$). IR ($cm^{-1}$) = 2924 (var, aliphatic CH stretch); 2854 (var, aliphatic CH stretch); 2103 cm-1 (str, azido); 1709 (str, C=O stretching); 1414 (var, C-O-H in plane bend); 1270 (var, dimer C-O stretch); 907 (str, O-H out of plane bend), in agreement with the literature [3].

## Fluorescein labelling of 1-adamantanamine

1-Adamantanamine (24 mg, 0.152 mmol) was dissolved in $CHCl_3$. Fluorescein isothiocyanante isomer I (FITC) was then added (6 mg, 0.0152 mmol for partial, low intensity labelling) and the mixture was stirred vigorously for 2h. The solid was filtered and exhaustively washed with $CHCl_3$ to remove unreacted FITC. The product obtained (orange solid) was examined with $^1$H NMR spectroscopy

(DMSO-$d_6$, 500 MHz): δ 7.8-6.9, m, and 6.6 (fluorescein), 2.09 (br s), 1.8 (br s), 1.671.5 (d, $J$ = 12 Hz)), 1.58 (d, $J$ = 12 Hz) (adamantane), corresponding to a fluorescein content of ~10%.

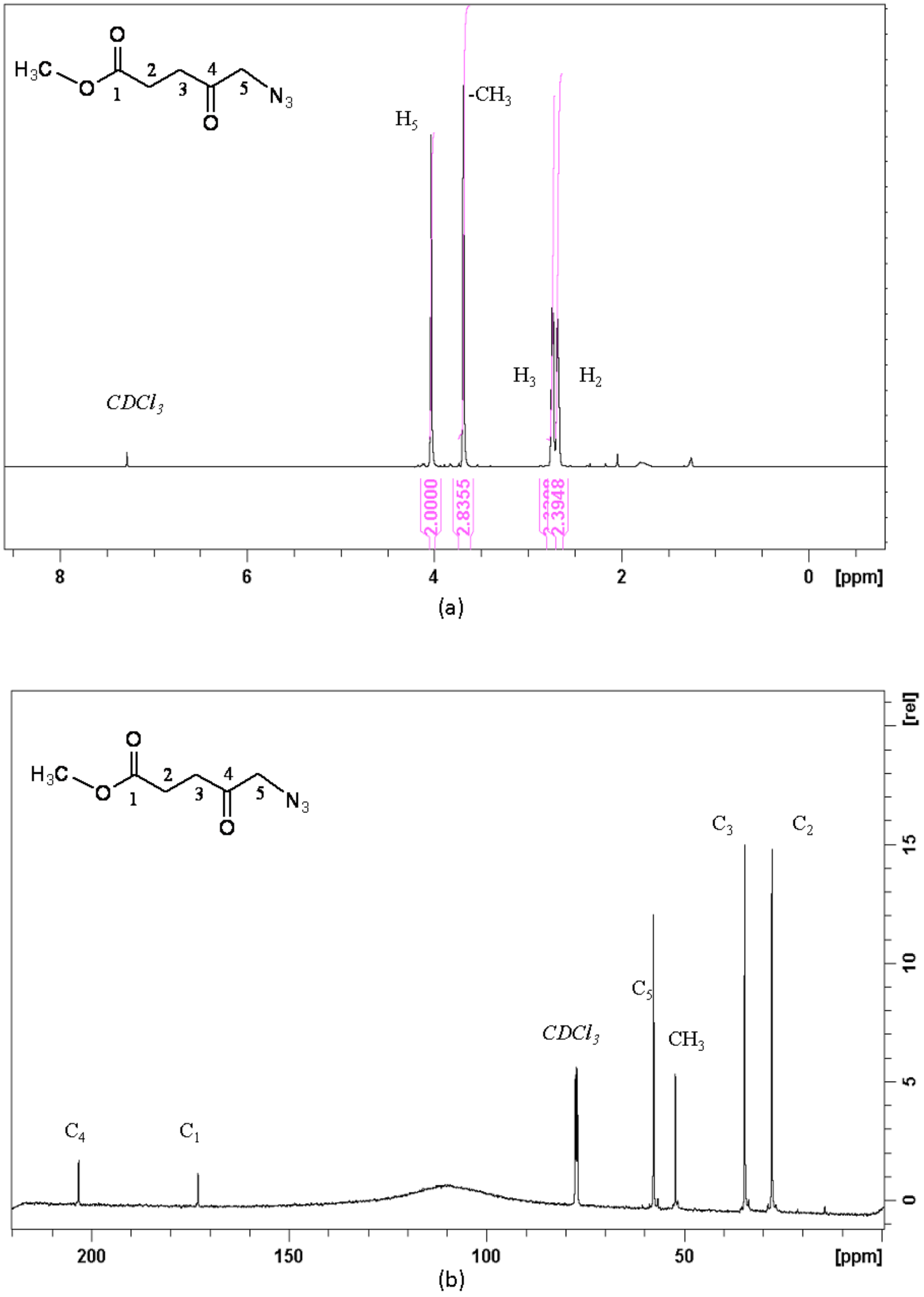


**Figure S1:** (top) $^{1}H$ (500 MHz, $CDCl_3$, $25^{0}C$) and (bottom) $^{13}C$ (125 MHz, $CDCl_3$, $25^{0}C$) NMR spectra of **7.**

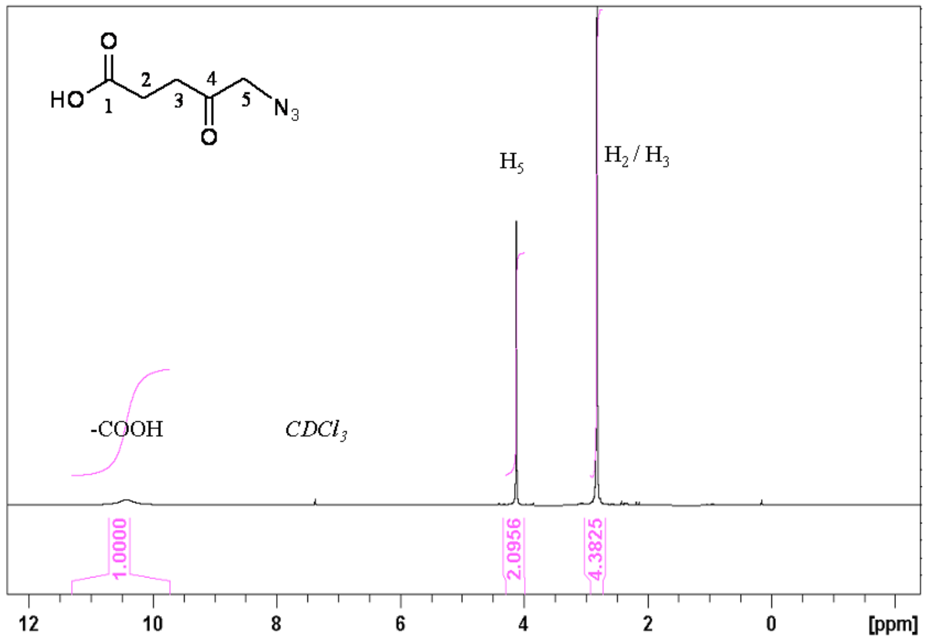


(a)

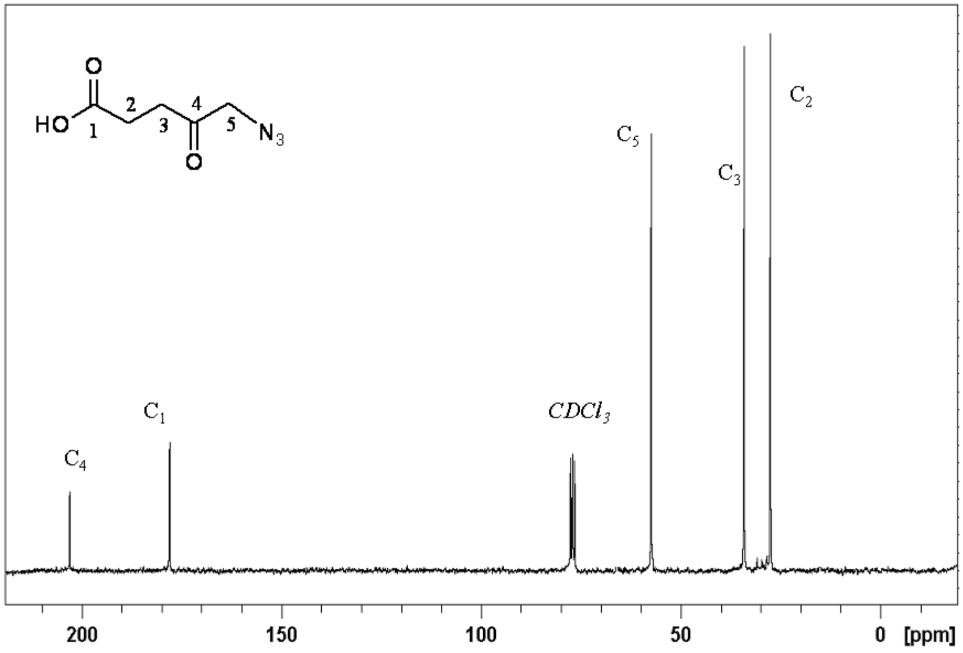


(b)

**Figure S2:** (top) $^{1}H$ (500 MHz, $CDCl_3$, 25$^{0}$C), and (bottom) $^{13}C$ NMR spectra of **8** (62 MHz, $CDCl_3$, 25$^{0}$C).

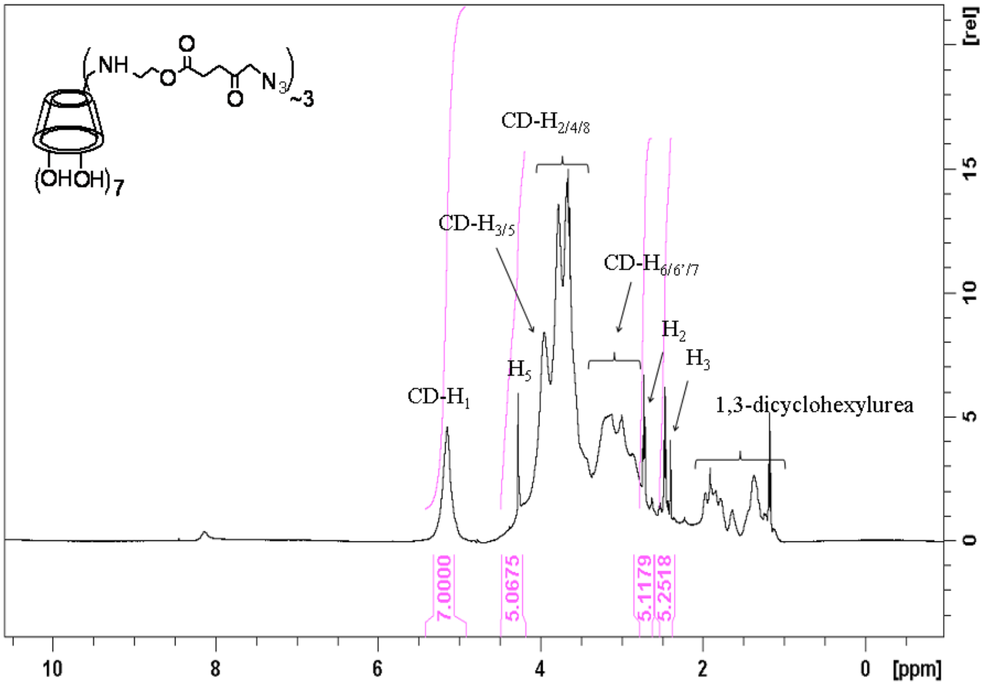


(a)

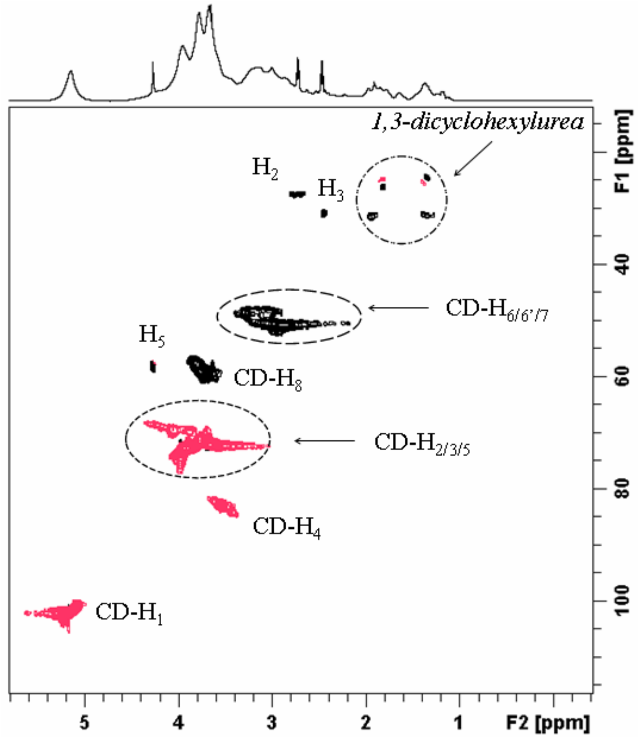


(b)

**Figure S3:** (top) $^1$H and (bottom) 2D HSQC-edited NMR spectra of **9** (500 MHz, $D_2O$, 25$^0$C).

(β)

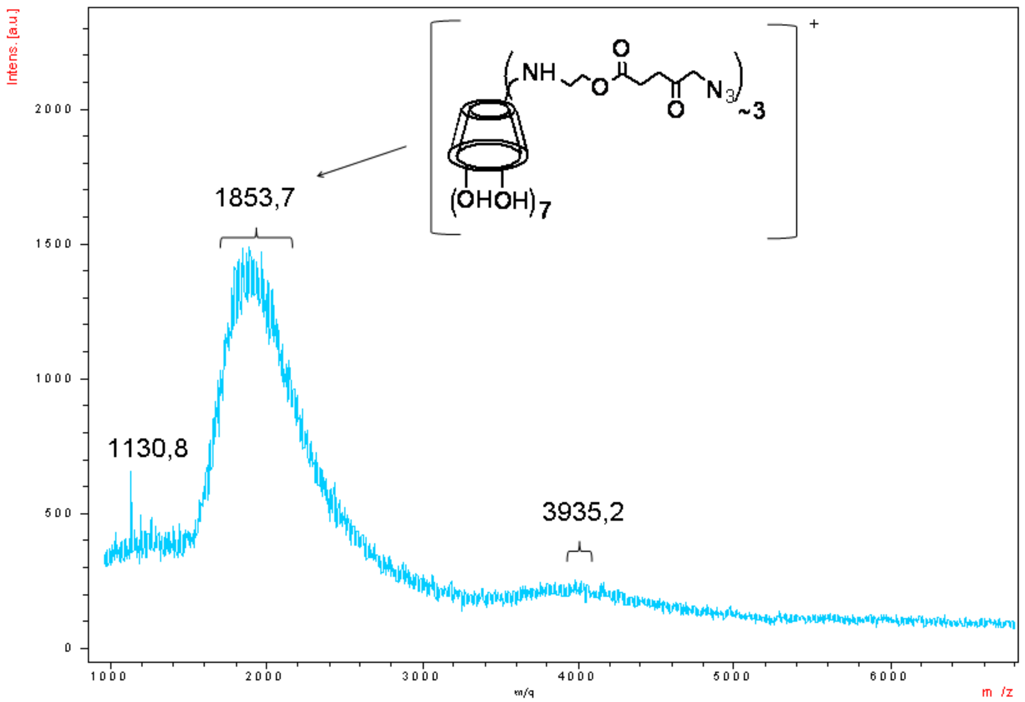


**Figure S4:** MALDI-TOF MS of **9** (Autoflex, Bruker Daltonics, positive polarity, linear mode).

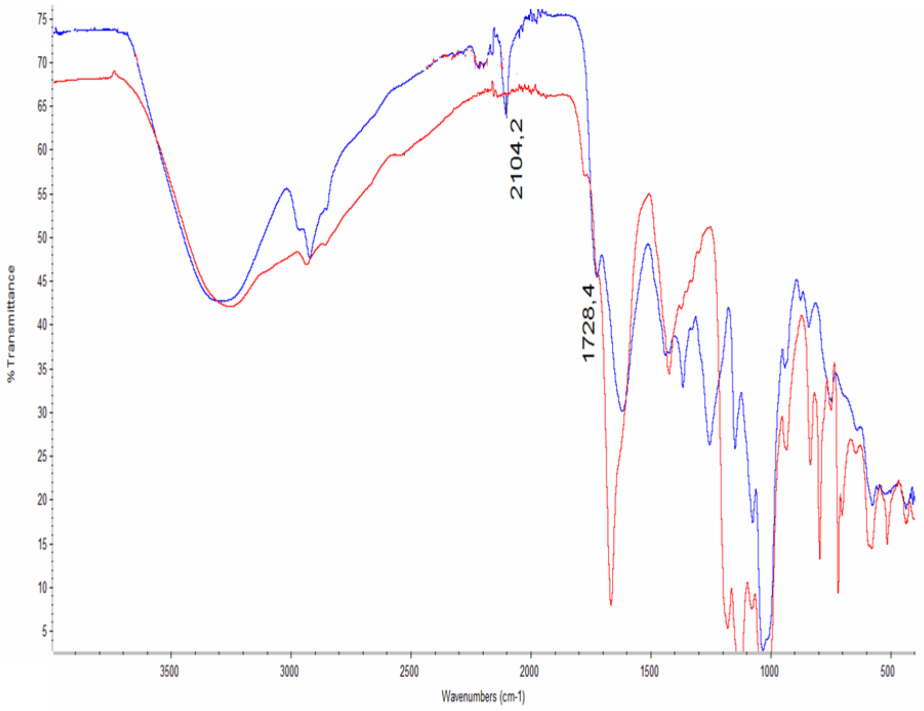


**Figure S5:** Overlay of FT-IR spectra of **9** (blue trace) and **9** (red trace) (ATR mode, solid sample).

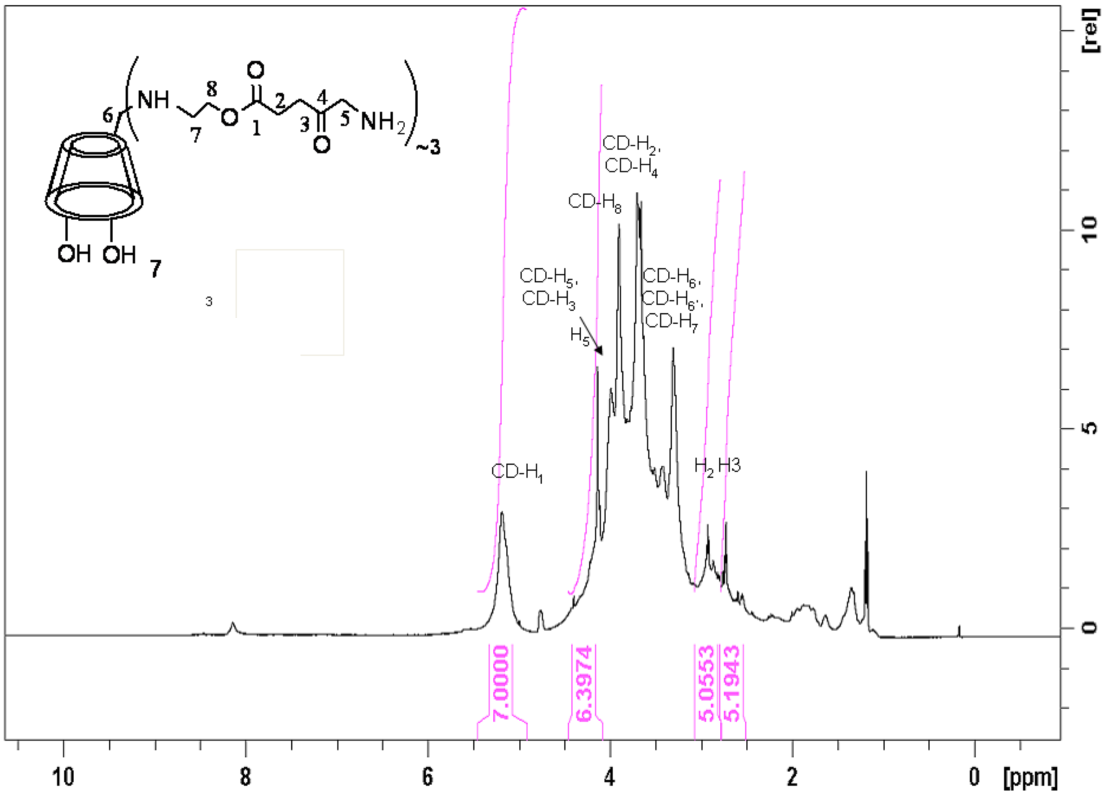


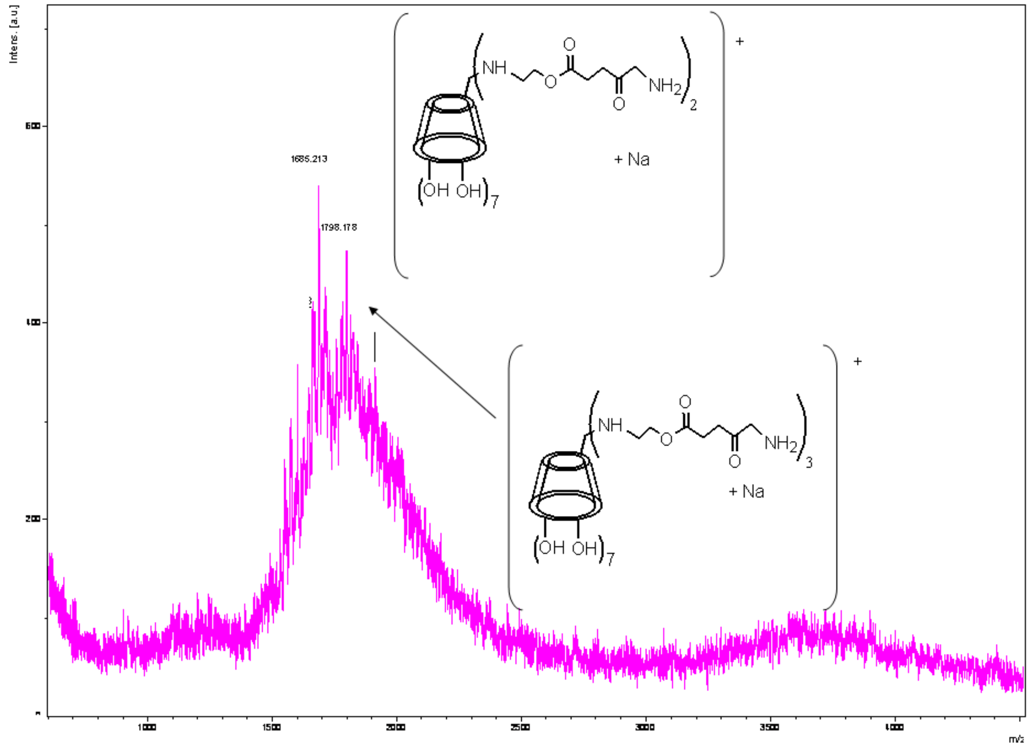


**Figure S6:** Conjugate **2**: (top) $^{1}H$ (500 MHz, $D_2O$, $25^0C$), (bottom) MALDI-TOF MS (Autoflex, Bruker Daltonics, linear mode, positive polarity).

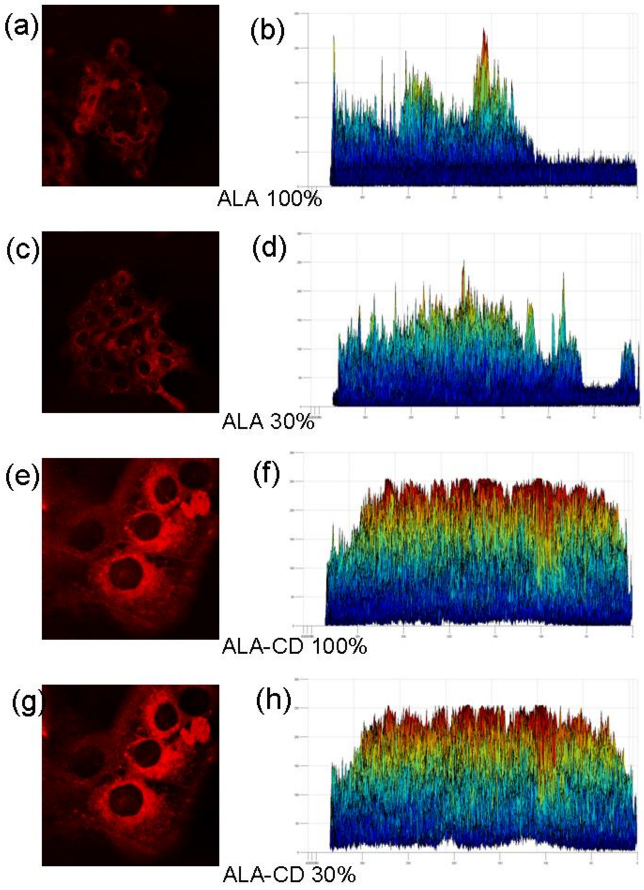


**Figure S7:** Representative confocal microscopy images of MCF7 cells ($\lambda_{ex}$=568 nm, $\lambda_{em}$ > 585 nm) incubated with 1 mM **1** (a: 100% laser intensity; c:30 laser intensity) and 1 mM **2** (e:100% laser intensity; g: 30% laser intensity), and the respective 2D MATLAB analysis of the red intensity and superimposition of 1D images along the *x*-axis. The color code: blue = lowest intensity; red = highest intensity.

# References


1. Vizitiu, D.; Walkinshaw, C. S.; Gorin, B. I.; Thatcher, G. R. *J. Org. Chem*. **1997,** *62*, 8760-8766.
2. Darcy, R.; O'Keeffe, F.; Schwinté, P. *J. Incl. Phenom. Macrocycl. Chem.* **1996**, *25*, 43–46.
3. Vallinayagam, R.; Bertschy, H.; Berger, Y.; Wenger, V.; Neier, R. *Synthesis* **2007**, *2007*, 3731-3735.
